# Properties of Water Confined in Ionic Liquids

**DOI:** 10.1038/srep10619

**Published:** 2015-05-29

**Authors:** Koji Saihara, Yukihiro Yoshimura, Soichi Ohta, Akio Shimizu

**Affiliations:** 1 Department of Environmental Engineering for Symbiosis, Soka University, 1-236 Tangi, Hachioji, Tokyo, 192-8577, Japan; 2 Department of Applied Chemistry, National Defense Academy, 1-10-20 Hashirimizu, Yokosuka, Kanagawa, 239-8686, Japan

## Abstract

The varying states of water confined in the nano-domain structures of typical room temperature ionic liquids (ILs) were investigated by ^1^H NMR and by measurements of self-diffusion coefficients while systematically varying the IL cations and anions. The NMR peaks for water in BF_4_-based ILs were clearly split, indicating the presence of two discrete states of confined water (H_2_O and HOD). Proton and/or deuterium exchange rate among the water molecules was very slowly in the water-pocket. Notably, no significant changes were observed in the chemical shifts of the ILs. Self-diffusion coefficient results showed that water molecules exhibit a similar degree of mobility, although their diffusion rate is one order of magnitude faster than that of the IL cations and anions. These findings provide information on a completely new type of confinement, that of liquid water in soft matter.

Room temperature ionic liquids (ILs) composed solely of bulky organic cations and anions exhibit useful properties, such as negligible vapor pressures, high ionic conductivities, flame resistance and thermal stability^1^,^2^,^3^,^4^. These substances are also recognized as potential designer solvents since their properties may be tuned by varying the cation/anion combination. It has also been suggested that ILs feature structural nano-heterogeneity^5^,^6^,^7^,^8^. Lopes and Pàdua^8^ simulated the structure of typical imidazolium-based ILs, and demonstrated the existence of a polar domain consisting of the imidazolium ring and the anion, and a nonpolar domain composed of the alkyl chains, representing an inhomogeneous nano-domain structure. These nanometer-sized domains are evidently proportional to the alkyl chain length of the imidazolium cation^9^. Subsequently, Jiang *et al.*^10^ reported results for the structures in 1-octyl-3-methylimidazolium nitrate ([OMIM][NO_3_])/water mixtures as determined by molecular dynamics (MD) simulations. The nano-domain structure of the original IL were found to be maintained even in mixed solutions in which water molecules were situated near the boundaries between the polar and nonpolar domains. Since then, additional information concerning the structure and dynamics of IL/water mixtures, focusing on the micro environment of both water molecules and IL cations/anions, has been obtained through various experiments^11^,^12^,^13^,^14^,^15^,^16^. In addition, we have recently succeeded in demonstrating “confined water,” or water-pockets, in the water-rich regions of mixtures of ILs with 80 mol% water, using small-angle X-ray (SAXS) and neutron scattering (SANS) analyses^17^. The confinement of water has been found to occur in both chemical and biological systems, including meso-porous silica materials and cell-like structures such as reverse micelles, in which the water structure or state near the interface is significantly altered compared to that in the bulk state. Confined water is therefore an important factor in a number of processes and there is significant interest within the scientific community in describing water structures and dynamics in various confined environments^18^,^19^. For this reason, it would be helpful to better understand the state of water when mixed with ILs. However, despite a large amount of research to date, direct observations of the state of water confined in ILs and an understanding of the states at the molecular level are still lacking. This is partly because the study of the structural and dynamic properties of disordered systems represents a very complex task due to the lack of suitable experimental methods.

In this study, we have succeeded in directly investigating the state of water locally situated in ILs by NMR spectroscopy. NMR is a powerful means of accomplishing this investigation since it provides information concerning individual atoms that reflect inter- and intra-molecular interactions. It is normally challenging to obtain sufficiently intense spectra for ILs in aqueous solutions, due to the large water peak that results from the dynamic range effect. The use of D_2_O allows NMR spectra of the IL to be obtained, but does not allow analysis of the water peaks by ^1^H NMR. As a means of resolving the above dilemma, we selected H_2_O+D_2_O mixed solution, for use in these trials. As an added benefit, the use of HOD may allow one to follow the H/D exchange reaction between H_2_O and D_2_O occurred inside the water pocket, which in a normal sense cannot be resolved on the NMR timescale, because the exchange is very fast in neat water. Additionally, by the use of infrared and Raman spectroscopies as a complementary technique to ^1^H NMR, Yaghini *et al.*^20^ pointed out that the effect of water on the local structure and phase behavior of imidazolium-based ILs is dependent on the particular cation-anion pair investigated. In this manner, we may expect that the equilibrium property of water molecules confined in ILs can be evaluated. The structures of the 1-butyl-3-methylimidazolium ([BMIM]^+^) and 1-ethyl-3-methylimidazolium ([EMIM]^+^) cations, both of which have exchangeable C2 ring protons in the imidazolium cation with water, and the *N*,*N*-diethyl-*N*-methyl-*N*-(2-methoxyethyl) ammonium ([DEME]^+^) cation investigated in this study are shown in Fig. 1. The corresponding ^1^H NMR spectra together with the spectrum of pure H_2_O/D_2_O mixture are also shown. The respective peaks were assigned by previous reports^21^,^22^,^23^. In each trial, the water was prepared by mixing H_2_O and D_2_O in different molar ratio.

## Results and Discussion

**Figure 2:**
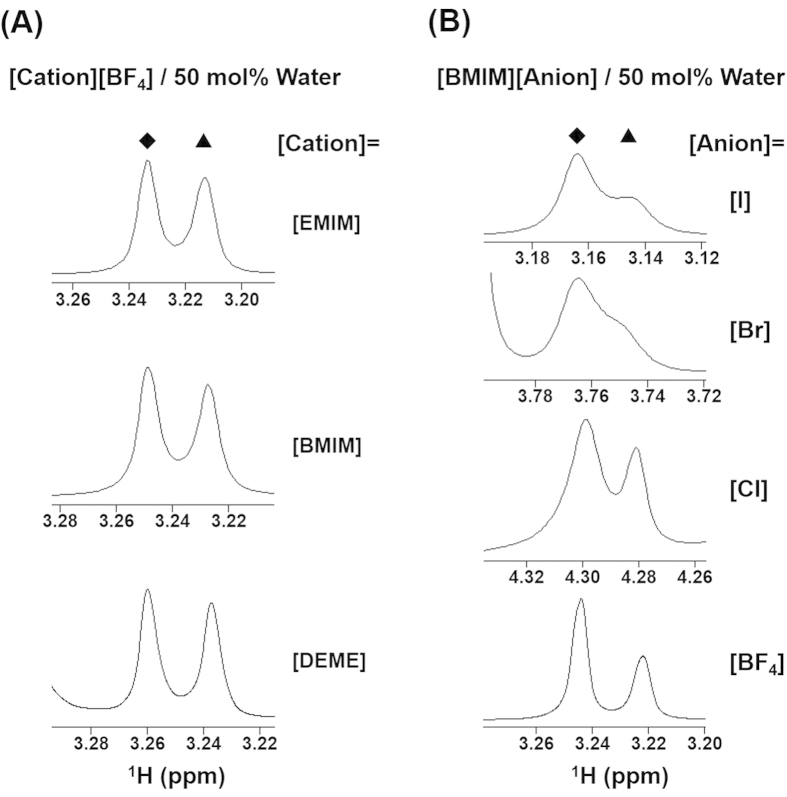
^1^H NMR spectra of (A) 50 mol% water mixtures of BF_4_-based ILs [DEME][BF_4_], [BMIM][BF_4_] and [EMIM][BF_4_] with HOD and (B) BMIM-based ILs [BMIM][Anion] (Anion = BF_4_, Cl, Br, I) in buffered solutions (adjusted to the pH 8 ∼ 9). Legends: ◆= de-shielded water, ▲= shielded water. ^1^H NMR chemical shifts, *δ*, relative to a tetramethylsilane (TMS) internal standard are shown. Typical ^1^H NMR measurements were conducted using an ECA-500, employing double NMR tubes to avoid the mixing sample and rock solvent containing the reference. Rock solvent containing 1 vol% TMS as the reference was placed in the inner tube (3 φ), which was then inserted into the outer tube with the sample.

**Figure 3:**
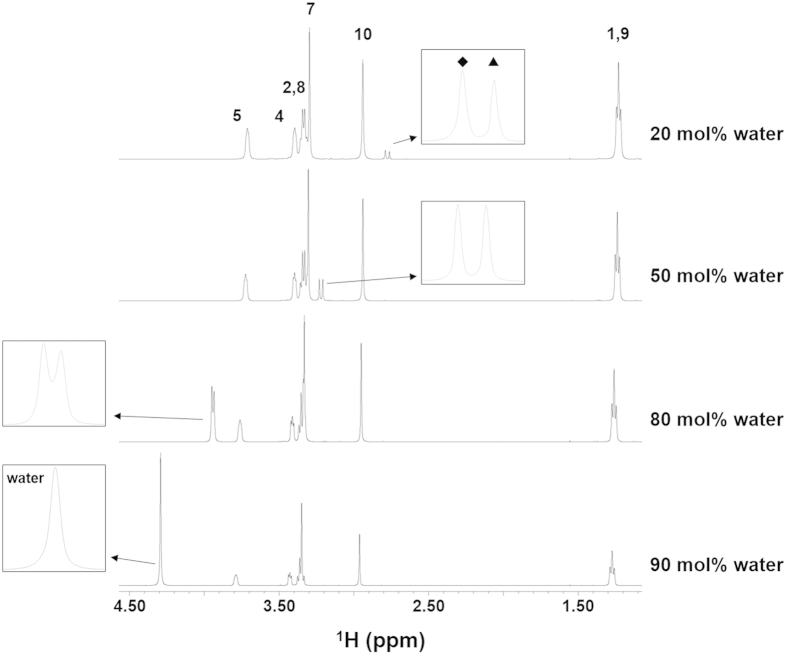
Typical example of the results for variations in the ^1^H NMR spectrum of [DEME]/water mixtures with differing water contents. HOD (H_2_O:D_2_O=1:1) was used as water. Legends: ◆ = de-shielded water, ▲= shielded water. Peak numbers correspond to the numbering of the carbon skeletons of the [DEME]^+^ cation shown in Fig. 1.

A typical ^1^H NMR spectrum obtained for a [DEME][BF_4_]/50 mol% water (H_2_O : D_2_O = 1 : 1) mixture is shown in Fig. 2(A). Remarkably, the peak resulting from the water protons is shifted to a higher magnetic field compared to that of pure water and is clearly split, indicating two different water states are present in the magnetic environment. A similar phenomenon was also observed in the other ILs investigated (Fig. 2(A)), and this splitting of the water peak could be observed even one week after preparation of the samples. The chemical shift from the spectrum of pure HOD was 4.807 ppm. It is well known that a decrease in the chemical shift is associated with reduced hydrogen bonding in the three-dimensional hydrogen bonded network water structure^24^. Thus, it appears that these mixtures exhibit significant microheterogeneity upon the addition of water to the IL. This may be due to the different environments experienced by the water protons as a consequence of confinement in the nano-domain structures of the ILs. The same splitting of the water spectrum was observed when varying the water concentration over a wide range, from 10 to 80 mol% (Fig. 3).

Here, it should be noted that equilibrium will exist among H_2_O, D_2_O and HOD in pure water, as below.
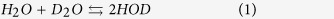


**Figure 4:**
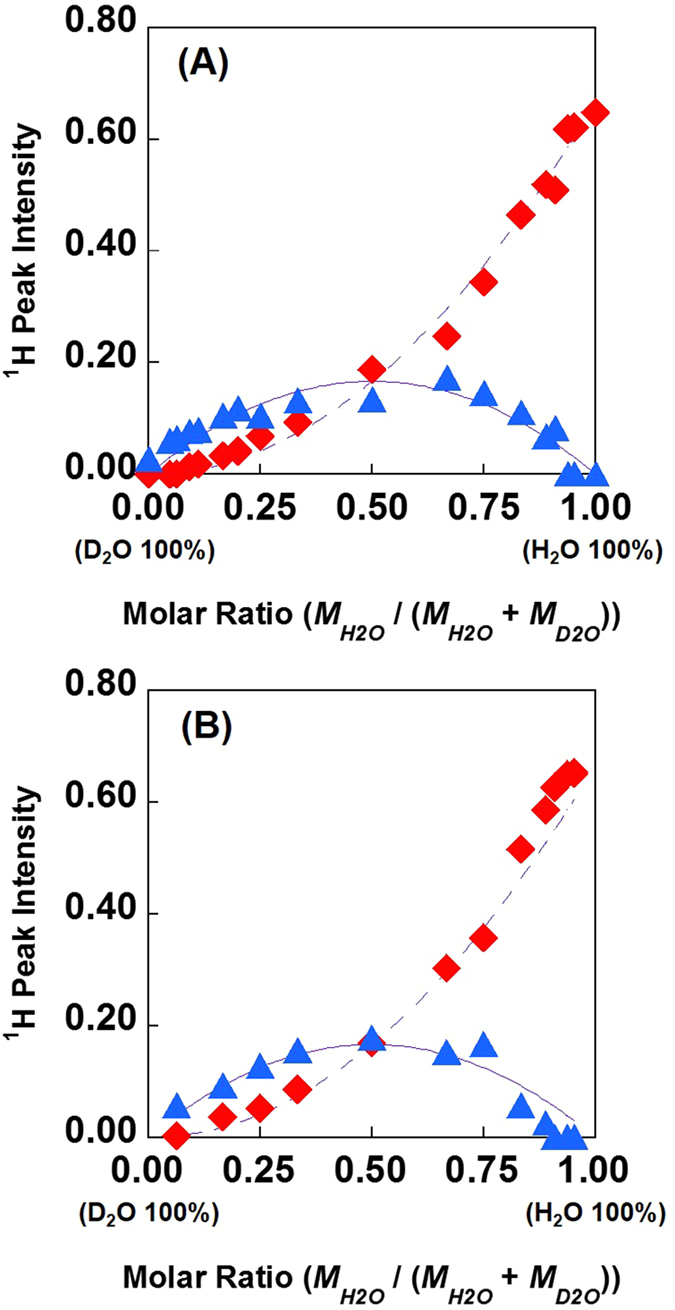
Peak intensity changes of the water peaks in the ^1^H NMR spectrum of (A) [DEME][BF_4_] and (B) [BMIM][BF_4_]/50 mol% water mixtures as the H_2_O:D_2_O molar ratio is varied in a 50 mol% water mixture. Legends: 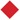
= de-shielded water peak intensity, 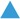
= shielded water peak intensity, dashed line = theoretical value of total H_2_O peak intensity, solid line = theoretical value of total HOD peak intensity. Note that H/D exchange reaction between the exchangeable proton and water does not occur in this condition (pH4.88-5.73).

As a result of this equilibrium, equilibrium constant *K* of water is,
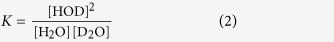


It is known that the combination of the water follows a binomial distribution by mixing H_2_O and D_2_O in any molar ratio^25^. Thus, H_2_O, D_2_O, and HOD were exist in our system. The above splitting water peak might correspond to H_2_O and HOD molecules. We attempted to ascertain whether the water peaks truly come from different species, by varying the H_2_O : D_2_O molar ratio in mixtures in which the water content was held constant at 50 mol% water (Fig. 4). Very interestingly, the area intensities of shielded and de-shielded water were completely consistent with the theoretical HOD line and the theoretical H_2_O line, respectively.

**Figure 5:**
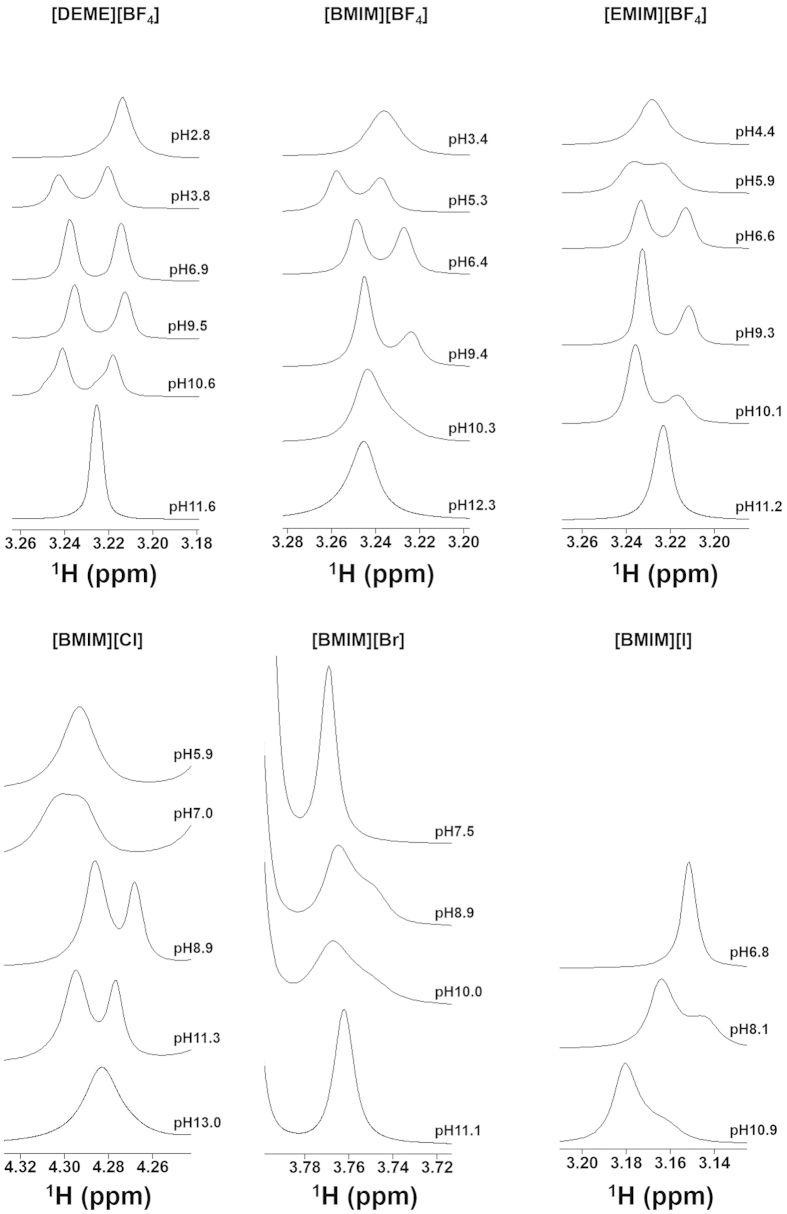
Variations of water peaks in the spectra of IL/ 50 mol% water mixtures with differing pH value.

**Figure 6:**
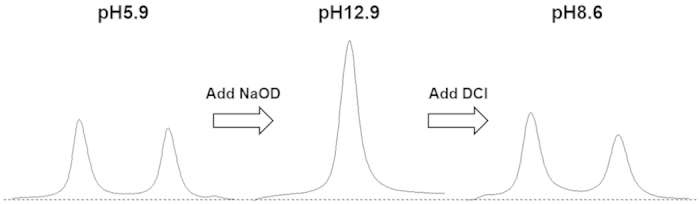
Variation in the spectrum of [DEME][BF_4_]-50 mol% water mixture with changing pH value.

If the shielded and the de-shielded water peaks in Fig. 4 correspond to HOD and H_2_O, *K* does not change in any H_2_O/D_2_O mixing ratio. Also, [D_2_O] can be calculated from the binomial distribution. The *K* determined from these value using equation (2) was [DEME][BF_4_]; 4.19 ± 0.67 and [BMIM][BF_4_]; 3.87 ± 0.82. Interestingly, this *K* value is good agreement with that for pure water (20% H_2_O/ 80% D_2_O), 3.86, reported by Duplan *et al.*^25^. In general, we treat the molecular or electrolyte solution as a homogeneous system, and as a result the water peak exhibits a beautiful single shape on the ^1^H NMR spectrum due to a very fast proton exchange rate among the water molecules. Thus, the results demonstrate that the proton and/or deuterium exchange rate between the H_2_O and the HOD molecule was abnormally slow in the water-pocket. Then, it may be possible to control the splitting of the water peak by varying the pH. We therefore ascertained the pH dependence of water peaks in the NMR spectra of IL/water mixtures (Fig. 5). As expected the water peak exhibited the single peak at strongly acidic and alkaline conditions. The resulting data also showed that, in the [DEME][BF_4_], [BMIM][BF_4_] and [EMIM][BF_4_] mixtures, peak splitting was observable over a wide range of pH, whereas the [BMIM][Br] and [BMIM][I] mixtures only exhibited a shoulder under these conditions. Furthermore, the separation width of the water peak was slightly different depending on the type of IL but showed essentially no change with variations in pH. For more supporting information, the variation in the water spectrum with changing pH cyclically is shown in Fig. 6. As is clear from the results, the water peak splitting reverts to the original one even from once strongly alkalized solution. Thus, these observations are consistent with the above described scenario. In this way, the peak splitting of water was observed in many kinds of ILs having aprotic cation and hydrophilic anion in this study. Importantly, no significant changes were seen in the chemical shifts obtained from the BF_4_^−^-based ILs with changes in the cation, although the chemical shifts varied greatly depending on the type of anion, as shown in the Fig. 2(B). It is therefore evident that the water within the ILs, i.e. proton exchange rate, is affected by the anion rather than the cation. Here, it interesting to quote that the distribution of water molecules around the BF_4_^−^ anion is much more unstructured and less site-specific, likely due to the strong interaction between the cations and anions^26^. In this IL, it has been suggested that the preferred location of the water molecules is at the apex of the tetrahedral BF_4_^−^ anion.

**Figure 7:**
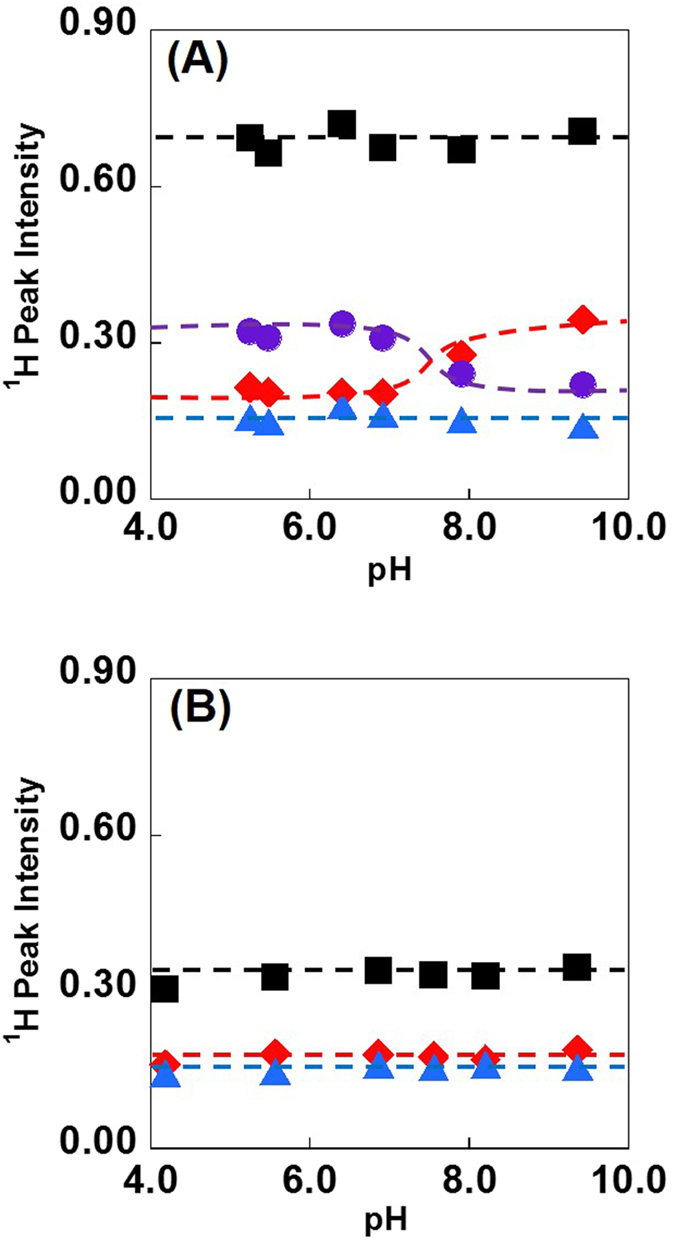
The ^1^H NMR peaks for (A) [BMIM][BF_4_] and (B) [DEME][BF_4_]/water mixtures at various pH values. The exchange of the deuterium in water with the C2-H of the imidazolium cation was determined by monitoring the decay of the C2-H proton. The deuterium exchange results in the disappearance of the signal due to the C2-H proton and the appearance of that due to H_2_O. Legends: 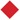
= de-shielded water peak intensity, 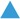 = shielded water peak intensity, 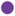 = C2-H proton peak intensity and ■ = total sum of the preceding peak intensities. The dashed lines are only guides for the eyes.

At this point, it is worth noting that an H/D exchange reaction occurs between the C2-protons of the imidazolium cations and D_2_O^27^,^28^, and more detailed information on the water present in the specific water-pocket may be deduced from an analysis of this reaction. For this reason, we subsequently examined the H/D exchange reaction between the C2-H protons of the imidazolium cations and HOD in a [BMIM][BF_4_]/50 mol% water mixture, a sample for which the water NMR peak was clearly separated. Fig. 7(A) summarizes the changes in the ^1^H integration of each peak for [BMIM][BF_4_]/water mixtures as the solution pH values are varied. The reaction does not proceed to any significant extent over a span of three days at a pH of 6.03. The H/D exchange reaction does take place above a pH of approximately 8, a result that is in agreement with previous data acquired from D_2_O mixtures^29^, since the intensity of the H_2_O peak increases as the C2-H peak intensity decreases. Interestingly, the intensity of the HOD peak remains constant. The sum of the C2-H peak, H_2_O peak and the HOD peak integrals are constant as the pH is varied. These results therefore unequivocally indicate that the overall mass balance is unchanged and that the varying intensity of the H_2_O peak under alkaline conditions is caused solely by an increase in the exchange rate. If this is true, the reverse reaction should not proceed even when the mixture is acidified after the exchange is fully completed under the alkaline conditions. As expected, we found that acidifying a solution with an initial pH of 13 to pH of 4 did not change the C2-H peak integration. Thus, the H/D exchange reaction is affected by the solution pH rather than by the reaction time period. For understanding the above results, we consider again the equilibrium in eq. (1). If [HOD] is constant, [H_2_O] increases with reducing [D_2_O] by the H/D exchange reaction between the C2-H and D_2_O, where *K* should take a constant value. In fact the *K* value in Fig. 7(A) was determined to be 3.73 ± 0.28. To further assess the above results, the H/D exchange reaction was investigated in [DEME][BF_4_]/water mixtures, since the [DEME] cation does not have an exchangeable proton. The results obtained from a [DEME][BF_4_]/50 mol% water mixture are shown of water in Fig. 7(B). In contrast to the results for the [BMIM][BF_4_] mixtures, none of the peak intensities changed with variation in the solution pH.

**Figure 8:**
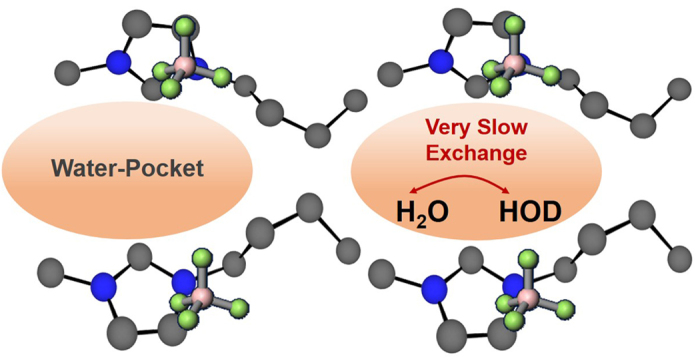
A schematic representation of the two waters in [BMIM][BF_4_]- 50mol % water.

It is helpful to understand the self-diffusivity of both the water molecules and the IL ions in these mixed systems. To the best of our knowledge, however, no experimental values have been reported for the self-diffusion coefficients, *D*, in [BMIM][BF_4_]/water mixtures. The literature value for the self-diffusion coefficient of pure H_2_O and HOD (that is, an equi-molar mixture of H_2_O and D_2_O) is 2.25 × 10^−9^ m^2^s^−1^ and 2.11 × 10^−9^ m^2^s^−1^ at 298 K^30^. The *D* values for the two species of water, i.e. H_2_O and HOD, in [BMIM][BF_4_]/50 mol% water mixtures were 4.39 × 10^−10^ (H_2_O) and 4.33 × 10^−10^ (HOD) m^2^s^−1^ respectively, and thus are nearly equivalent, indicating that the water molecules in both states experience a similar environment, in which their diffusivity is approximately one order of magnitude slower than in bulk water. Molecular dynamics (MD) simulations^10^,^31^ have shown that water clusters, such as dimers and trimers, inside a water pocket behave differently from bulk water, and thus the data from the present experiments are in good agreement with these simulations. The *D* values obtained for the IL cation and anion were 6.63 × 10^−11^ and 7.08 × 10^−11^ m^2^s^−1^, respectively. One order of magnitude lower than those determined for the two water species. The results obtained concerning the confinement of water in the IL nano-domain structures can be explained by considering that the stable polar/nonpolar networks formed in the IL due to the strong interactions between cations and anions are only minimally disrupted by the addition of water. In previous studies, we found that the chemical shifts of the ^1^H signals generated by the [BMIM] cation exhibit very little change up to a water concentration of 90 mol%^29^. Zhong *et al.*^26^ suggested that the IL ions are somehow able to slightly adjust their relative position/orientation when water is inserted into the IL structure. The results in the present study may also give a hint to explain why the nearly-free hydrogen bonds (NFHB) state of water in the [BMIM][BF_4_]/water^32^ and [DEME][BF_4_]/water^33^ systems is preserved even at 77 K, at which temperature a hydrogen bonded network would be expected to form, as in neat H_2_O. Finally, these pictures proposed here are shown in Fig. 8.

For more information, we need to be aware of recent studies by Yaghini *et al.*^34^ who reported the effect of water on the transport properties of ILs for protic (1-ethyl-3-methylimidazolium) and aprotic (1-ethylimidazolium) ILs cations with less hydrophilic trifluoromethanesulfonate (TfO) to more hydrophilic bis(trifluoromethanesulfonyl)imide (TFSI) anions in view of the analyses of the self-diffusivity, conductivity, and proton exchange mechanism. Overall, the *D* values of ions are one order magnitude lower than those of water in entire water concentration investigated, which is in agreement with our present results. The difference in the *D* values for the ionic species was observed depending on the combination of cations and anions. Water molecules tend to interact with different sites in the protic and aprotic ILs because a strong preference for hydrogen bonding to the –NH group and a stronger affinity to the TfO anion. Thus the proton exchange between the –NH group and D_2_O water for the protic cation with TfO anion occurs at a faster rate as compared to the TFSI anion^34^. The results suggest that the polarity of ILs is important as a structure parameter in predicting the water coordination^20^.

In summary, we proposed a simple but smart probe, HOD, to obtain good quality NMR spectra of representative ILs / water mixture in order to investigate the state of water in such systems. Our work applied NMR to detect the presence of water in two different species, i.e. H_2_O and HOD, in mixtures of water with various ILs. These water molecules were found to be situated in close proximity to one another, and to be loosely confined inside the IL framework. Proton exchange rate between the water molecules showed abnormal slowness in water-pocket sited nano-domain structure, and consequently water peak split into two. Such signals are highly sensitive to the anion but rather insensitive to the cation, thus allowing a discrimination between domains within ILs. Further discriminating properties are the different pH response of the two signals and the different exchange ability towards C2-H of imidazolium.

It is well known that water is a unique liquid that exhibits many anomalous properties. ILs are also very unique liquids, having numerous unexpected characteristics. Intriguingly, simply mixing these two unusual liquids presents the opportunity to obtain water in multi-species confined in various ILs, as the IL cations and anions make slight adjustments in their relative positions and orientations to accommodate water molecules. The results presented herein provide new insight into the physicochemical properties of water in the nano-domain structures that are a key of characteristic of ILs, and may also assist in elucidating the structures and dynamics of water in various confined environments and/or complex systems.

## Methods

Six typical ILs were selected: 1-butyl-3-methylimidazolium tetrafluoroborate (usually abbreviated as [BMIM][BF_4_]), [BMIM][Cl], [BMIM][Br], [BMIM][I], 1-ethyl-3-methylimidazolium tetrafluoroborate ([EMIM][BF_4_]) and *N*,*N*-diethyl-*N*-methyl-*N*-(2-methoxyethyl) ammonium tetrafluoroborate ([DEME][BF_4_]), all of which have been well studied and for which basic properties information is readily available. These were all purchased from Kanto Chemical Co., Inc. and used without further purification. The NMR spectra of these ILs were also obtained and assessed to ensure that no impurities were present. Both H_2_O and D_2_O were ultrapure and were supplied by a Synergy UV system (MILLIPORE Inc.) and by Kanto Chemical Co., Inc. (99% D), respectively. Mixtures of different concentrations (mol% water) were prepared by combining the required amounts of the IL and water.

The pH values of the mixed solutions were adjusted as required by the addition of either NaOD or DCl solutions (10-, 100-, 500- and 1000-fold dilutions). During such adjustments, care was taken to avoid changing the concentration of the original mixture by more than 1%. The pH values of the mixed solution were assessed using a pH electrode (CEM Co. CM055-BNC) and pH meter (HORIBA, Ltd. D-52).

NMR studies were carried out using an ECA-500 (JEOL 500 MHz) and an AVANCE III HD (Bruker 600 MHz). The DOSY (Diffusion-Ordered NMR Spectroscopy) measurements were performed by applying a 2 ms magnetic field gradient pulse width and a 3 mT/m to 0.24 T/m magnetic field gradient strength. The ^1^H DOSY parameters were as follows: 15.0 ppm spectrum width, 64 K sample points, 8 to 20 s pulse delay (the relaxation times of the ILs and water were 0.29 to 4.4 s), 8 scans, 0.114 Hz digital resolution (0.000228 ppm) at 296 ± 0.1 K. Similarly, the ^19^F DOSY parameters were the following: 20.0 ppm spectrum width, 16 K sample points, 8 to 20s pulse delay, 8 scans, 0.575 Hz digital resolution (0.00122 ppm) at 296 ± 0.1 K. Accuracy of the measurements was less than 3% standard error in the diffusion study. Spectral analysis was performed using the Delta NMR software package of the JEOL RESONANCE or commercially available processing software developed in Visual Basic Ver. 6 (Data Processing of FT-NMR by PC, Second edition, Sankyo Publishing, 2009). A Lorentzian-Gaussian mixed function was employed for the analysis.

## Additional Information

**How to cite this article**: Saihara, K. *et al*. Properties of Water Confined in Ionic Liquids. *Sci. Rep.* **5**, 10619; doi: 10.1038/srep10619 (2015).

## Figures and Tables

**Figure 1:**
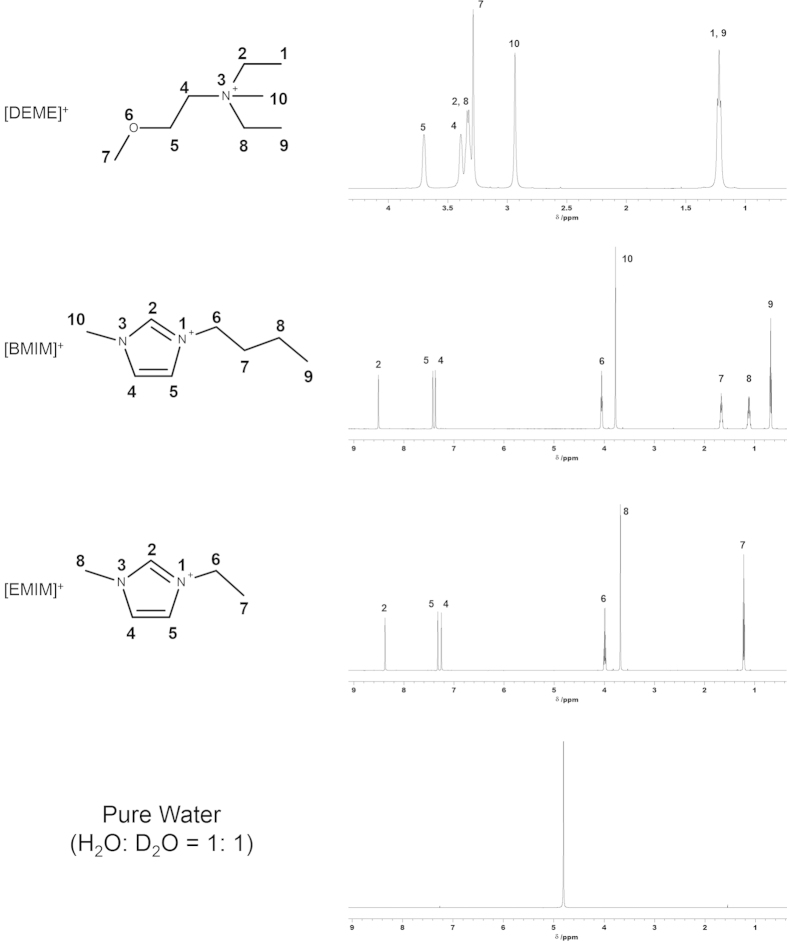
Structures of *N*,*N*-diethyl-*N*-methyl-*N*-(2-methoxyethyl) ammonium ([DEME]^+^), 1-butyl-3-methylimidazolium ([BMIM]^+^) and 1-ethyl-3-methylimidazolium ([EMIM]^+^) cations. The corresponding ^1^H NMR spectra of [DEME][BF_4_], [BMIM][BF_4_], and [EMIM][BF_4_] along with the pure H_2_O/D_2_O mixture (in molar ratio of 1 : 1) are also provided. The numbering of the carbon skeletons of the cations is also shown.
